# A Reliable Low-Latency Multipath Routing Algorithm for Urban Rail Transit Ad Hoc Networks

**DOI:** 10.3390/s23125576

**Published:** 2023-06-14

**Authors:** Lei Suo, Liu Liu, Zhaoyang Su, Shiyuan Cai, Zijie Han, Haitao Han, Feng Bao

**Affiliations:** 1 School of Electronic and Information Engineering, Beijing Jiaotong University, Beijing 100044, China; 2 Traffic Control Technology Co., Ltd., Beijing 100071, China

**Keywords:** ad hoc, urban rail transit, multipath routing, routing maintenance

## Abstract

With the advancement of urban rail transit towards intelligence, the demand for urban rail transit communication has increased significantly, but the traditional urban rail transit vehicle–ground communication system has been unable to meet the future vehicle–ground communication requirements. To improve the performance of vehicle–ground communication, the paper proposes a reliable low-latency multipath routing (RLLMR) algorithm for urban rail transit ad hoc networks. First, RLLMR combines the characteristics of urban rail transit ad hoc networks and uses node location information to configure a proactive multipath to reduce route discovery delay. Second, the number of transmission paths is adaptively adjusted according to the quality of service (QoS) requirements for vehicle–ground communication, and then the optimal path is selected based on the link cost function to improve transmission quality. Third, in order to enhance the reliability of communication, a routing maintenance scheme has been added, and the static node-based local repair scheme is used in routing maintenance to reduce the maintenance cost and time. The simulation results show that compared with traditional AODV and AOMDV protocols, the proposed RLLMR algorithm has good performance in improving latency and is slightly inferior to the AOMDV protocol in improving reliability. However, overall, the throughput of the RLLMR algorithm is better than that of the AOMDV.

## 1. Introduction

Urban rail transit is steadily increasing its position in the field of public transportation because of its large capacity, fast speed, and high safety. With the deep integration of new information technologies and urban rail transit, such as Big Data, Satellite Communication, and Block Chain, the demand for urban rail transit communication is growing rapidly, and achieving a more intelligent, safe, efficient, and economical urban rail transit system is the future development direction [1]. In this paper, the main research is how to improve the network communication performance of urban rail transit vehicle–ground communication systems with multiple services.

The traditional urban rail transit generally used communication-based train control (CBTC), which has the advantages of small departure intervals and high operating efficiency, but with the increasing demand for operation efficiency, the existing vehicle–ground communication system is unable to meet the communication requirements in the future [2]. The network architecture design of vehicle–ground communication systems is an important research object for the next-generation urban rail transit. At present, the urban rail vehicle–ground communication network adopts WLAN or LTE-M technology to carry CBTC, PIS, CCTV, and other services [3]. In this wireless communication network, there are a large number of network facilities, such as the need to set up multiple pieces of trackside equipment and base stations at the trackside; the base stations are connected to the station’s network switches, and the terminals need to establish connections to the core network through the base stations to complete the communication with the station. Therefore, this network architecture is complex to build and costly to operate and maintain, and when the central node is disturbed or damaged, the vehicle–ground communication will be broken, which will cause problems such as emergency braking, delayed running, and the stopping of trains. For this reason, it is difficult to meet the demand for low-latency and high-reliability vehicle–ground communication in the next-generation urban rail transit system. There are many ways to improve the performance of vehicle–ground communication. Reference [4] balances energy usage and passenger appearance time by considering the joint optimization of passenger and energy allocation, thereby improving the performance of train–ground communication. Reference [5] focuses on train scheduling and proposes three optimization models for train scheduling to improve the efficiency of train–ground operations. This article focuses on the impact of wireless communication on vehicle–ground communication.

There has been research on distributed communication network technology for urban rail transportation. Based on the design idea of the “Starlink Project” [6], the researchers propose a communication scheme for applying ad hoc network technology to an urban rail transit communication network, which is called “Rail Starlink”, which removes the ground facilities, upgrades the trackside equipment to communication nodes, and enables direct communication among the nodes through the reasonable placement of communication nodes. Finally, the urban rail transit ad hoc networks composed of mobile nodes, trackside nodes, and sink nodes are realized to carry out urban rail transit communication services. Compared with the traditional urban rail wireless communication network, the urban rail transit ad hoc network has the following characteristics:

(1) Decentralized network. Add a routing function in the trackside equipment and use a Device-to-Device (D2D) interface without intermediate node control forwarding to realize the direct communication between the vehicle–ground and trackside equipment
(2) Lower cost. It does not need to rely on ground facilities, reducing the construction and maintenance costs of base stations and core networks, and vehicle and trackside equipment use wireless communication, with no need to deploy and maintain cables.
(3) Lower latency. The vehicle–ground and vehicle–vehicle communication do not transmit data through basic network facilities but rather transmit data through trackside equipment in multiple hops, thereby reducing network latency.
(4) Stronger robustness. The ad hoc network does not need a central node to control and coordinate the operation of the network and has strong invulnerability. When a node or link fails, it will repair itself or replace the path, so the failure will not affect the operation of the overall network.

In recent years, ad hoc networks as a new network communication technology have been widely used in the fields of vehicular ad hoc network (VANET), wireless sensor network (WSN), flying ad hoc network (FANET), and satellite communication. Among them, routing protocols are an important part of network communication technology.

Traditional routing protocols can be divided into proactive routing and reactive (on-demand) routing based on different routing strategies, and it can be divided into flat routing and hierarchical (clustering) routing according to the network topology. According to the number of paths used, it can be divided into unipath routing and multipath routing [7].

Domestic and foreign researchers have studied routing protocols mainly from the perspectives of energy, network load, link stability, and routing maintenance according to the characteristics of ad hoc networks in different fields. Ref. [8] proposed EDAODV and AODV-I protocols, where EDAODV uses a bi-directional path discovery mechanism to bypass congested nodes; the AODV-I protocol adds congestion handling to Route Request (RREQ) to avoid selecting congested paths and also adds a route repair mechanism to improve the congestion problem. A multipath strategy for the link lifecycle as well as link energy loss prediction, which effectively reduces energy consumption and latency by selecting paths with long link survival and low energy consumption, was provided in [9]. In [10], a heuristic Q-Learning algorithm was proposed for the problem of frequent network topology changes and poor link stability in VANETs, which adaptively adjusts the parameter values through continuous interaction with the external environment to find a good stable path, improving the reliability of packet delivery. In [11], to address the problem that traditional routing repair incurs huge routing overhead in terms of energy and delay, an efficient routing recovery protocol with the endocrine cooperative particle swarm optimization algorithm (ECPSOA) was proposed, which uses multi-swarm evolution equations to improve the rate of convergence and accuracy of the repair algorithm and reduces routing overhead and energy consumption. To overcome the problem of link instability in VANET, a hybrid multipath routing protocol with intelligent decisions is proposed, which can find routes quickly and uses intermediate nodes for failure maintenance, thus reducing the average routing latency and improving reliability [12]. Reference [13] proposes a method based on node residual energy and link stability, which modifies the original AODV routing protocol and adds header information on node residual energy and stability to the RREQ routing request message, enabling the selection of nodes with high stability and sufficient residual energy during the routing discovery process. Reference [14] proposes a new unmanned aerial vehicle-assisted urban VANET (VRU) based on the AI algorithm. This algorithm includes two sub-protocols for routing data and supporting the detection of malicious vehicles: the VRU_Vu protocol for data packet transmission between vehicles with the assistance of drones and the VRU_u protocol for data packet transmission between drones.

In summary, there are many studies related to ad hoc network routing protocols combining the characteristics of WSN networks and VANET networks, but few studies of routing algorithms focus on ad hoc networks for urban rail transit. In the urban rail transit ad hoc network, the network nodes need to be used for a long time and have a long-term power supply system, so there is no need to consider the loss of node energy, and the network nodes are randomly distributed around the trackside, which differs from VANET networks in that they contain many static trackside nodes. So, the existing routing algorithm cannot give full advantage to the characteristics of urban rail transit networks to improve the network communication performance. Considering the multipath routing technology, it not only enhances the link fault tolerance but also relieves network congestion, which improves the reliability of data transmission and the transmission latency and improves the QoS of urban rail communication services. Consequently, it is important to study the multipath routing algorithm of urban rail transit ad hoc networks based on low-latency and high-reliable communication.

The main purpose of this article is to provide a routing algorithm suitable for urban rail transit ad hoc network scenarios, namely, the reliable low-latency multipath routing algorithm (RLLMR). First, based on the ad hoc network scenario of urban rail transit, a static configuration routing strategy is proposed to shorten the time for on-demand routing discovery. Second, in response to the issue of different communication requirements for different services in the process of urban rail transit vehicle–ground communication, a multipath routing algorithm that can adaptively adjust parallel routing based on services requirements is proposed to meet the latency requirements of different services as much as possible while saving routing resources. Finally, a local routing repair scheme based on maintenance nodes is added to enhance the reliability of the communication process by introducing a routing maintenance mechanism. This method has a faster repair speed compared to traditional backup routing repair methods and can reduce maintenance costs. In addition, the performance of RLLMR was compared with that of AODV and AOMDV, as shown in Table 1.

The rest of this paper is organized as follows. Section 2 introduces the urban rail self-organizing network model. Section 3 discusses the multipath selection routing algorithm and the route repair scheme. Section 4 shows the simulation results. Finally, Section 5 summarizes this paper.

## 2. The Network Model

In ad hoc networks, the common network structure is a planar network topology, whose characteristics include a high density of network nodes; all nodes have the same function, nodes can cooperate to complete the work, which has better robustness, and the node load is more balanced.

In a flat network, using traditional proactive routing protocols requires all network nodes to maintain global routing information for a long time and to update routing information at a time when the network topology changes, so this requires a significant maintenance overhead. Using on-demand routing protocols, the route discovery process requires the multiple-forwarding of RREQ messages, resulting in a lot of time being consumed, which seriously affects the transmission efficiency.

**Figure 1:**
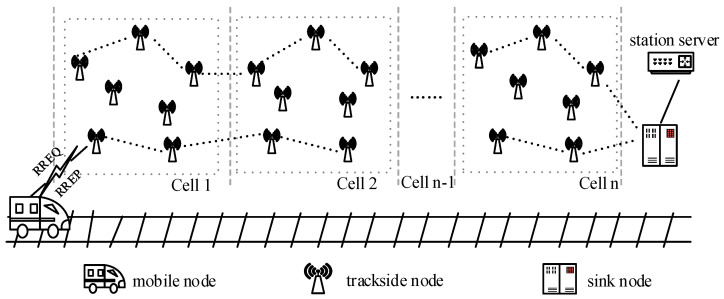
The network topology of the train and trackside nodes.

In this paper, combining the characteristics of an urban rail transit ad hoc network, the network is divided into several areas based on the maximum communication range of nodes, some trackside nodes are distributed in each area, and the routing information is stored in the routing table of trackside nodes in advance through using the characteristics of stationary trackside nodes, so the intermediate nodes have the routing information to reach the station sink nodes, and each node only needs to maintain the routing information of their respective paths, thus reducing the routing overhead. Finally, the network topology of the train and trackside nodes is shown in Figure 1.

In the urban rail transit ad hoc network, the trackside nodes are static and linearly distributed around the trackside, the train nodes move along the tracks dynamically, and sink nodes are concentrated in the stations. In Figure 1, it can be seen that there are mobile nodes, trackside nodes, and sink nodes in the network, and each type of node has different ways of working. The functions and working modes of each node are as follows.

(1) Mobile Nodes. This type of node essentially refers to the wireless terminal located at the front or rear of the train, which combines routing and control management functions. First, this type of node has the ability to aggregate the respective business data within the train carriages. Second, it is responsible for sending information to ground facilities. This type of node has a relatively small number in the network and is mainly responsible for carrying out various services of vehicle–ground communication. In the process of vehicle–ground communication, routing discovery is performed first, followed by data forwarding.
(2) Trackside Nodes. It can also be called the relay node. This type of node is numerous, and different trackside signaling facilities can all serve as trackside nodes after adding routing functions. This type of node is statically distributed near the trackside in a linear pattern in the network, and the trackside nodes can be powered by underground cables. Therefore, there is no need to consider the issue of insufficient node energy during the research process. During the static configuration phase, routing information such as ground sink nodes and some special nodes will be written in advance in the routing table. In the process of vehicle–ground communication, the main role of trackside nodes is to forward data information from adjacent nodes and achieve multi-hop communications.
(3) Sink Nodes. It can also be called the gateway node. This node is mainly located next to the station, and it is mainly responsible for sending and receiving data information forwarded by the mobile nodes or trackside nodes and, finally, transmitting the data to the control server of the station through fiber, Ethernet, and other basic network facilities.

## 3. The Improved Multipath Routing Algorithm

The routing algorithm is improved mainly to optimize the latency and packet loss rate and to enhance the communication performance of the network system. In this section, first, the characteristics of urban rail transit ad hoc networks are analyzed, and it is explained that configuring static routes can improve the route discovery algorithm; then, with a suitable mathematical equation, it is explained that the method of constraining the number of paths according to different service latency requirements and selecting paths based on the link cost can improve the transmission efficiency and reliability; finally, in order to improve the reliability of communication and address the issues of high repair costs and long repair times in traditional route maintenance schemes, a local route repair scheme is discussed.

The following notation is used for formulating the proposed approach.
General variables and parameters*N*The number of available paths.*n*, *m*The total number of sent packets.*i*Available path index, *i* = 1, 2, …, *N*.*j*, *k*Index per hop, representing from the (*k* − 1)-th node to the *k*-th node, *j* = 1, 2, …, $hc_{i}-1$, *k* = 1, 2, …, $hc_{i}-1$.*M*The number of parallel paths, *M* = 1, 2, …, *N*.*hop*(*i*),
$hc_{i}$The number of hops from the source node to the destination node for the *i*-th available path.*x*The hop count.*y*The link quality.$L_{R}$Control message size, such as RREQ and RREP.*L*Transmission packet length.$T_{queue}$The queue time of each hop.$T_{propagation}$The propagation time of each hop.$\bar{T_{d2d}}$The average end-to-end latency.$T_{f}$The latency of route discovery.$T_{r}$The routing repair latency.*T*The longest latency of transmitting packet data in *M* paths.$T^{\prime }$Retransmission latency.*B*The transmission bandwidth.*f*The carrier frequency.*q*Urban rail service index, *q* = 1, 2, …$\tau_{q}$Requirements for the transmission latency of urban rail services.*λ*The average amount of data arriving in unit time.*μ*The average amount of data processed in unit time.*p*, *P*,
$P_{s2d}$The packet loss rate of data transmission.$P_{loss\_rate}(j)$The packet loss rate of the *j*-th node.TH(i)Throughput of the *i*-th path.$P_{L}$The path loss.$P_{t}$The transmitting power.$P_{t}$The received power.$G_{t}$, $G_{r}$The antenna gain.*R*Communication range.

### 3.1. Adaptive Multipath Selection Algorithm Based on Service Requirements

As a variety of services need to be carried out in the urban rail transit vehicle–ground communication system, a unipath routing algorithm is used for data transmission, which cannot meet the communication needs of different services. on the one hand, and on the other hand, network congestion easily happens, which causes data loss and affects the efficiency and reliability of the vehicle–ground communication system. Therefore, how to meet the communication needs of different services is an important issue.

Multipath routing technology has the features of providing different paths for communication services with different QoS requirements and providing multiple paths for data of the same communication services. So, using multipath routing algorithms, it is possible to flexibly carry out several services and also meet the communication requirements of different services. However, traditional multipath routing algorithms, such as the AOMDV, take a lot of time to flood RREQ messages in the network during route discovery. So, the algorithm proposed in this paper addresses the above problem and speeds up the route discovery. Generally, end-to-end delay includes transmission delay, processing delay, queuing delay, and propagation delay. Existing routers are generally high-speed routers with the processing latency of microseconds or even lower, so it is unnecessary to consider it. In the process of route discovery, it is necessary to first send RREQ to the destination node and then reply to RREP to the source node; the latency of route discovery [15] is shown in Equation (1).

(1) $$T_{f}=2*hop(i)*(\frac{L_{R}}{B}+T_{queue}+T_{propagation})$$

in which *hop*(*i*) is the number of hops from the source node to the destination node, *i* represents the number of independent disjoint paths that are allowed to be discovered, $L_{R}$ is the control message size, *B* is the transmission bandwidth, $T_{queue}$ is the queue time of each hop, and $T_{propagation}$ is the propagation time of each hop.

In the route discovery process of the on-demand routing protocol, the source node needs to send RREQ to find the routing information of the destination node, and when the routing information is found, the node replies to the source node with a route reply (RREP) message, which completes the establishment of the link. Therefore, combined with Equation (1), it is known that the number of hops is the main influencing factor on the route discovery time. So, this paper proposes a static configuration routing scheme for urban rail transit networks. The scheme enables the source node to obtain the routing information of the destination node from the routing table of the intermediate node during the route discovery process to reduce the number of control message forwarding and decrease the route discovery latency.

Providing a different number of paths for different services can more fully utilize routing resources and relieve the network load effectively. In this paper, a multipath selection algorithm based on service requirements is proposed. The algorithm constrains the number of paths according to the service latency requirements and then selects transmission paths based on the link state. Now, assume that the nodes in the network have the same routing performance, and because the network range is small, when the mobile node runs at 120 km/h, the propagation delay is also in microseconds, so the transmission delay and queuing delay are mainly considered in the estimation of delay. The packet arrivals obey Poisson distribution. According to the knowledge of queuing theory, *λ* represents the average amount of data arriving in unit time, *μ* represents the average amount of data processed in unit time, and each link can be considered as a standard M/M/1 model. Thus, the average arrival rate and service rate with the multipath parallel transmission mode are *λM* and *μM*, and *M* is the number of parallel paths. The constraint Equation (2) is obtained according to the latency requirement.

(2) $$\tau_{q}>\alpha \sum_{k=1}^{{\max\{hc}_{i}\}}\left(T_{trans,k}+T_{queue,k}\right)$$

(3) $$T_{trans,k}=\frac{L/M}{B}$$

(4) $$L_{que}=\frac{\rho^{2}}{\left(1-\rho \right)}$$

(5) $$T_{queue,k}=\frac{L_{que}}{\lambda M}=\frac{\lambda }{(\mu -\lambda )\mu M}$$

where $\tau_{q}$ represents the latency requirement of different services, *q* is the service type, *α* is the estimation factor, which represents the maximum number of retransmissions, $\left\{hc_{1},hc_{2},\ldots ,hc_{i}\right\}$ is the number of hops corresponding to different paths, $T_{trans,k}$ represents the transmission delay of the kth node, $T_{queue,k}$ represents the queue delay of the kth node, *L* is the packet length, $L_{que}$ is the queue length, and ρ represents the queuing strength of the system, in which *ρ* = *λ*/*μ*. According to Equations (2)–(5), the minimum number of parallel links *M* (*M* should be less than the number of independent disjoint paths) can be calculated.

The average end-to-end latency of transmitting *n* packets using *M* paths is shown in Equation (6).

(6) $$\bar{T_{d2d}}=[n*(1-p)*T+p*n*\{T+T^{\prime }\}]/n$$

(7) $$T=\underset{M}{\max}\{\sum_{k=1}^{hc_{i}}\left(T_{trans,k}+T_{queue,k}\right)\}$$

where *p* is the packet loss rate of data transmission, and *T* represents the longest latency of transmitting packet data in the path.

The traditional AOMDV only considers one factor of hop count in the routing selection process, and it ensures transmission efficiency by selecting the least-hop-count path. However, this selection scheme cannot guarantee the quality of the propagation path while ensuring transmission efficiency. When selecting the bad-quality path to transmit data, even though this path has a few hop counts, it causes frequent packet loss because of the bad path quality and affects the transmission efficiency. So, in this paper, the hop count *x* and link quality *y* are chosen as the influencing factors to select a better path by establishing the link cost function. Among them, a larger parameter x means a longer path, which has a significant impact on the end-to-end latency and is negatively correlated with the reduction in latency; a larger parameter y means a good link quality, which is beneficial for improving the reliability, and it is positively correlated with the reduction in the packet loss rate. The link cost function is shown in Equation (8).

(8) $$LS(x,y)=\alpha *x+\beta *\frac{1}{y},\alpha +\beta =1$$

where *α* and *β* are weight coefficients, which represent the degree of contribution of the parameters to the delay and reliability, and it can be obtained according to the AHP algorithm. Then, according to the Dijkstra principle, select the M links with the lowest link cost as the transmission path.

The traditional AOMDV routing protocol will select one or more routes with the minimized hop count from the remaining paths as alternate routes after the completion of the primary path selection, and when a path fails, the node will generate a route errors (RRER) message and then inform the source node to enable the alternate path for retransmission; this way can improve the reliability of communication. In general, researchers use the packet loss rate as a measure of reliability, and the nodes can calculate the packet loss rate by counting the number of successfully received packets, as shown in Equation (9).

(9) $$P_{loss\_rate}(j)=1-\frac{receive\_nums(j)}{send\_nums}$$

(10) $$P_{success}=P(P_{t}-Los-P_{N}>\theta )=\frac{1}{2}\left\{1+erf(\frac{\psi (d)}{\sqrt{2}\sigma })\right\}$$

(11) $$\psi (d)=P_{t}-\theta +P_{N}+28-20\mathrm{lg}f-18\mathrm{lg}d$$

(12) $$Los=P_{L}+\xi ,\xi ∼(0,\sigma^{2})$$

where $P_{loss\_rate}(j)$ represents the packet loss probability of the *j*-th node ($j_{\max}$ means the destination node), *send_nums* is the number of packets sent from the source node, receive_nums(j) represents the number of successfully received packets at the *j*-th node, $P_{L}$ represents the path loss, and *ξ* represents the shadow fading coefficient, which follows a normal distribution with a standard deviation *σ*. The successful reception of packets by a node mainly depends on whether the received power can be higher than the received threshold after the influence of large-scale fading and noise power, and if it is higher than the received threshold, the reception is considered successful. The packet loss rate from the source node to the destination node can be estimated by combining Equation (9), as shown in Equation (13).

(13) $$P_{s2d}=[1-\sum_{M}\left(1-P_{loss\_rate}(j_{\max})\right)]*P_{loss\_rate}^{B}(j_{\max})$$

It can be seen that increasing the number of alternate paths *B* will result in more paths retransmitting packets, thereby improving the packet loss rate and increasing the reliability of the system.

In addition, by combining the above equations, the throughput of the path can be calculated as shown in Equation (14).

(14) $$TH(i)=\frac{L*(1-P_{loss\_rate}(i))}{\bar{T_{d2d}}}$$

where TH(i) represents the throughput of the *i*-th path, and $L*(1-P_{loss\_rate}(i))$ represents the number of bits received by the destination node. The throughput represents the average rate at which a data packet can successfully reach the destination node from the source node.

### 3.2. Local Routing Maintenance Scheme Based on Maintenance Nodes

In the traditional AOMDV, the route repair time is long with the alternate path repair scheme [16], and the alternate path will be idle for a long time when no failure occurs, which has a large maintenance cost. Therefore, combined with the characteristics of an urban rail transit network, a static configuration scheme is used to improve the routing maintenance scheme in the traditional AOMDV routing protocol, and a local routing maintenance scheme based on maintenance nodes for local repair is proposed.

**Figure 2:**
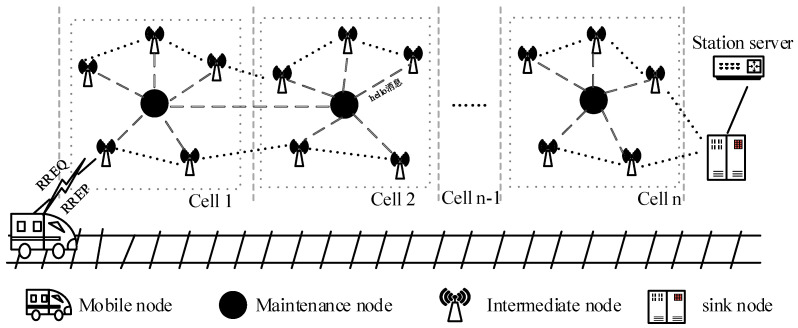
Vehicle–ground communication network architecture.

In the network model shown in Figure 1, the network area is divided into several virtual cells based on the area size and node location information; one or more maintenance nodes are arranged in each cell and configured with the routing information of the maintenance nodes, which reaches each network node in the current cell and reaches the maintenance node in the next cell. According to the network geographic area analysis, each maintenance node should be located in the center of the cell, and its position should satisfy the Equations (15) and (16).

(15) $$dis1=D(m_{j}(i)-\frac{1}{N}\sum_{N}m_{j}(i))$$

(16) $$dis2=D(\frac{1}{N}\sum_{N}m_{j}(i)-\frac{1}{N}\sum_{N}m_{j}(i+1))$$

in which *dis*1 and *dis*2 represent the Euclidean distances between the maintenance node and each other node in the current cell, the maintenance node in the current cell, and the maintenance node in the next cell, and $m_{j}$ represents the node position information in the *i*-th cell. The structure of the vehicle–ground communication network is shown in Figure 2.

It is seen from the figure that by configuring the routing information of the maintenance node and other nodes in advance, it is beneficial to find the routing information of the maintenance node quickly and complete the path replacement during the route repair process, which can avoid the problem of the long, time-consuming route repair scheme.

Considering whether the destination node can successfully receive the packet is related to the reception power of the node. So, in the route maintenance scheme, through monitoring the strength of the control signals sent by the nodes (such as Hello messages and Hello-ACK messages), it is possible to build a path loss model between two nodes, as shown in Equation (17).

(17) $$P_{L}(d)=P_{L}(d_{0})+10n\log_{10}(\frac{d}{d_{0}})$$

(18) $$P_{L}(d_{0})=10\log(\frac{P_{t}}{P_{r}})=-10\log[\frac{G_{t}G_{r}\lambda_{1}{}^{2}}{{\left(4\pi d_{0}\right)}^{2}}]$$

where $P_{t}$ is the transmit power, $P_{r}$ is the received power, $P_{L}(d_{0})$ is the path loss in free space, n is the attenuation factor, $G_{t}$ and $G_{r}$ represent the gain of transmitting and receiving antennas, $\lambda_{1}$ is the wavelength, $d_{0}$ is the reference distance, and *d* is the distance of two nodes.

With the known transmit power, the received signal strength of the next hop can be calculated based on the path loss model to determine whether it is lower than the set reception threshold so that the occurrence of a failure can be predicted. If the signal strength of the next hop is less than the set threshold, a failure is considered to exist, transmitting data to the next hop will fail, and a route repair algorithm needs to be initiated to replace the path. There are three repair cases depending on the location of the fault.

**Figure 3:**
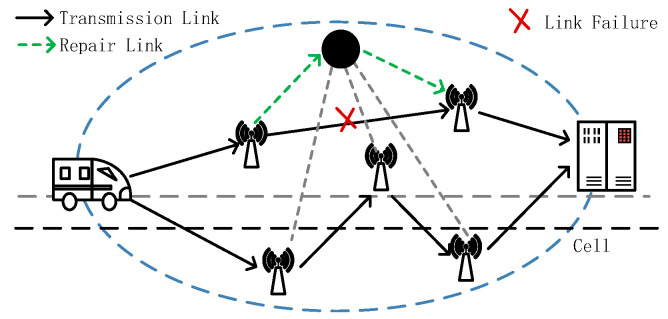
Intra-cell routing repair.

**Figure 4:**
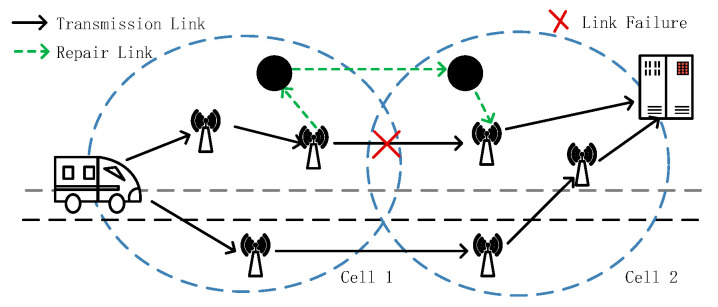
Inter-cell routing repair.

(1) Case 1: the established path is broken due to the mobile node moving beyond the communication distance, and data transmission cannot be successful. For this case, the mobile node restarts the route discovery algorithm to find a newly available path.
(2) Case 2: the data transmission fails due to the failure of the Intra-cell link. In this case, the node that discovers the failure sends an RRER message to the maintenance node in the area and establishes a temporary path with the help of the maintenance node, as shown in Figure 3.

(3) Case 3: the link failure happens between two cells, and the Intra-cell maintenance node cannot directly establish a temporary path for repair due to the limited communication range of the maintenance node. When the node detecting the failure finds that the next hop is not in the current cell, the node sends an RRER message to the maintenance node to let the maintenance node establish a connection with the maintenance node of the next cell to establish an available temporary path, as shown in Figure 4.

In summary, the routing repair scheme proposed in this paper predicts link failures by estimating the received signal strength of the nodes, and once a failure is found, it can be repaired quickly with the help of maintenance nodes. The average end-to-end latency using local routing repair schemes is shown in Equation (19).

(19) $$\bar{T_{d2d}}=\sum_{i}^{M}[\frac{m}{M}*(1-P)*\{\sum_{hc_{i}}T_{i}+T_{f}\}+\frac{m}{M}*P*\{T_{f}+\sum_{hc_{i}}T_{i}+T_{r}\}]/\frac{m}{M}]/M$$

where *m* is the number of packets transmitted, *P* is the probability of the link failure of each hop, $T_{i}$ represents the end-to-end latency of the *i*-th hop, $T_{r}$ represents the route maintenance time, and $T_{f}$ represents the route discovery time.

### 3.3. The Network Process

**Figure 5:**
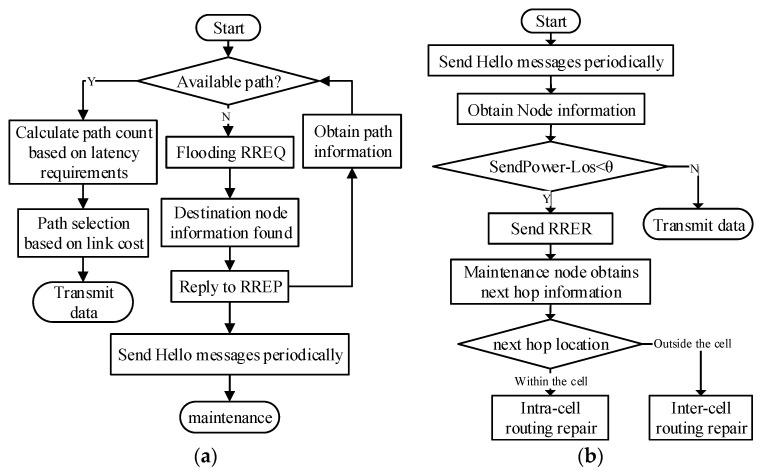
RLLMR algorithm flowchart (**a**) Routing Selection process. (**b**) Routing repair process.

The network process can be divided into six phases described in Table 2. The detailed processes are shown in Figure 5.

**Table 2:** Network process.

Stage	Function
Stage 1: Static configuration	Initialize the network nodes and configure multiple independent disjoint static paths with the position information of the nodes.
Stage 2: Routing discovery	The mobile node generates RREQ and floods RRE; when it finds the route information of the destination node, it sends an RREP message to the source node, and then the source node records the available path information after receiving the RREP messages and goes to the next stage.
Stage 3: Routing Selection	The number of parallel paths is calculated according to the latency requirements of the service data to be transmitted, and then, based on the available path information, it calculates each link cost and selects the path with the smallest cost as the primary path; the detailed process is shown in Figure 5a.
Stage 4: Data Transmission	The packets are transmitted along the primary path to the destination node, and during transmission, the intermediate nodes predict whether the next hop can be successfully received based on the transmit power and path loss model. If the failure message is received, it enters the route maintenance stage.
Stage 5: Routing Maintenance	The node generates an RERR message to send to the maintenance node after the maintenance node receives it, which will confirm the location information of the next hop based on the failure location, and then adopts the corresponding maintenance plan according to the location information; the detailed process is shown in Figure 5b.

### 3.4. RLLMR Algorithm

According to the above discussions, the proposed scheme is presented in Algorithm 1.
**Algorithm 1**. A reliable low-latency multipath routing algorithm (RLLMR)**Result:** Update routing table; 1: Initialize the routing table of network nodes 2: # Step 1: static routing configuration 3: Generate a virtual node to send RREQ 4: **for** intermediate node received RREQ **do** 5:    **for** without received the same RREQ-ID && new RREQ-ID > old RREQ-ID **do** 6:       record the IP of the source node in the reverse routing table 7:       send RREQ 8:    **end** 9: **end** 10: **for** destination node received RREQ **do** 11:    **for** destination node receives i RREQ-ID **do** 12:       add tag i to RREP 13:       reply to RREP along the reverse path 14:    **end** 15: **end** 16: **for** intermediate node received RREP **do** 17:    reply to RREP along the reverse path 18:    record the forward path in the static routing table 19: **end** 20: remove virtual nodes, there are a total of N static paths in the network 21: # Step 2: route selection 22: **for** mobile node sends data to destination node **do** 23:    **for** mobile nodes for routing discovery **do** 24:       send RREQ 25:       receive RREP 26:       record the discovered available paths x (x <= N) 27:       Get $hc_{i}$, y (from MAC layer), *λ*, *μ* 28:       **end** 29:    Find type of data sent q 30:    Get $\tau_{q}$, packet length *L* 31:    Calculate $\left\lceil M_{\min}\right\rceil =\frac{\alpha }{\tau_{q}}\sum_{{\max\{\mathrm{hc}}_{i}\}}\left(\frac{L}{B}+\frac{\lambda }{(\mu -\lambda )\mu }\right)$, Get *M* 32:    **for** estimating link cost **do** 33:       Calculate $LS_{i}(hc_{i},y)=\alpha *hc_{i}+\beta *\frac{1}{y}$ 34:    **end** 35:    **for** M > 0 **do** 36:       mark the available path with the minimum link cost as valid 37:       *M* − 1 38:    **end** 39: **end** **Update** mobile node routing table; 40: # Step 3: local routing repair 41: divide the network area into several areas according to R, mark cell 1.2…n 42: **for** intermediate node has detected a link failure reaching the next hop **do** 43:    Get current node location information, A($x_{1}$, $y_{1}$, $z_{1}$) 44:    Get next hop location information, B($x_{2}$, $y_{2}$, $z_{2}$) 45:    **for** node A and B are located in the same cell, n **do** 46:       mark the original path as a fault in the routing table 47:       mark the route to the maintenance node as valid 48:       send RRER to maintenance node, mark 1 49:    **end** 50:    **for** node A and B are located in the different cell **do** 51:       mark the original path as a fault in the routing table 52:       mark the route to the maintenance node as valid 53:       send RRER to maintenance node, mark 2 54:    **end** 55: **end** 56: **for** maintenance node received RRER **do** 57:    **for** mark = 1 **do** 58:       mark the route to the next hop as valid 59:    **end** 60:    **for** mark = 2 **do** 61:       mark the route to the maintenance nodes in next cell as valid 62:       send RRER to maintenance node, mark 1 63:    **end** 64: **end**

## 4. Performance Analysis of the Proposed Routing Algorithm

### 4.1. Simulation Environment Parameters

In this section, the main focus is on verifying the impact of the multipath selection algorithm and route repair scheme on the latency and reliability for urban rail transit vehicle-to-ground communication services. At first, the simulation is performed regarding the urban rail transit ad hoc network topology, which distributes trackside nodes in the network and makes the mobile nodes gradually approach the station sink nodes along a straight line, and then the proposed adaptive multipath routing algorithm (AMS) and local routing maintenance (LRM) scheme are compared with the traditional AODV [17] and AOMDV routing protocols. The simulation parameters are given in Table 3.

### 4.2. Analysis of Simulation Results

#### 4.2.1. Latency Results

The route discovery latency is the total time consumed by the source node in sending RREQ messages to find an available path to the destination node. The route discovery process of traditional AODV and AOMDV is compared with the route discovery process using a static configuration scheme, and the route discovery delay results are shown in the following figure.

**Figure 6:**
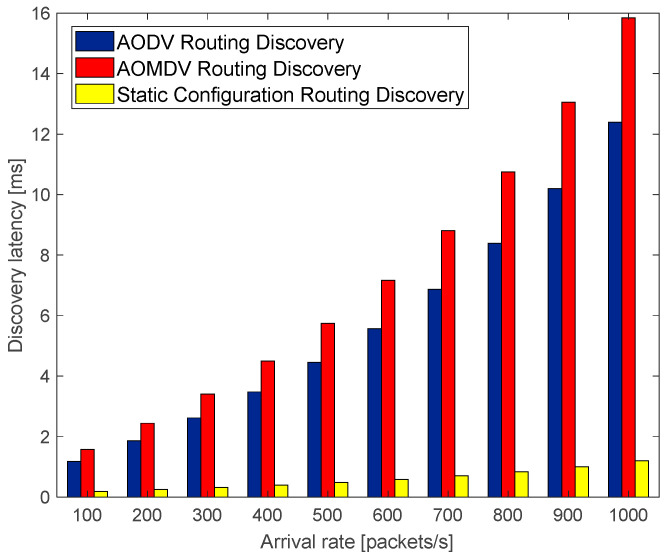
The relationship of discovery latency with arrival rate.

As shown in Figure 6, with the increase in the arrival rate, this means that, in unit time, the number of packets sent increases, which leads to a more congested network, and the route discovery latency increases exponentially. However, the route discovery latency with the traditional route discovery algorithm is affected by the arrival rate to a greater extent than with the route discovery algorithm under static configuration; that is because the traditional route discovery algorithm needs to multiple-forward RREQ messages, resulting in spending a long time finding the destination node routing information, while the route discovery algorithm based on the static configuration scheme can obtain the routing information of the destination node through intermediate nodes to effectively reduce the number of RREQ forwarding. Consequently, this method can greatly reduce route discovery latency.

**Figure 7:**
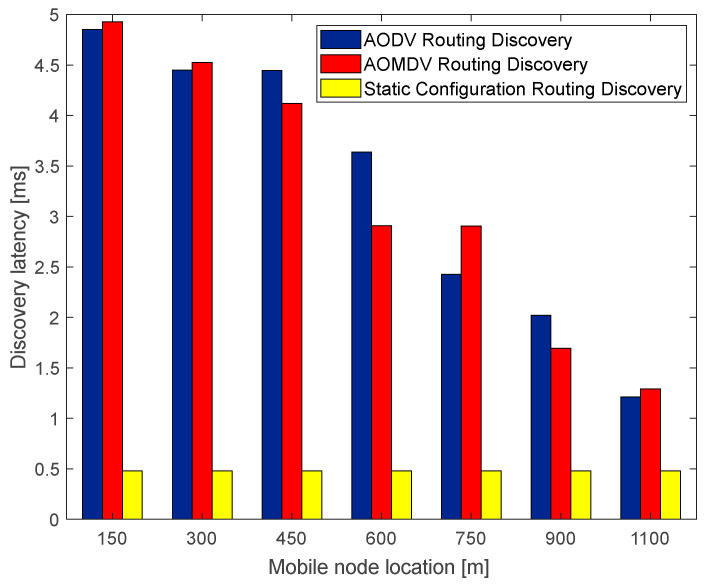
The relationship of discovery latency with mobile node location.

As shown in Figure 7, the AODV route discovery latency and the AOMDV route discovery latency fluctuate as the mobile node approaches the station, which is due to the change in the number of RREQ message forwarding, that is, the hop count to reach the destination node during the movement of the node. It shows that the hop count is the main influencing factor for the route discovery latency. Therefore, configuring static route information can effectively reduce route discovery latency by reducing the number of route discovery request forwarding.

**Figure 8:**
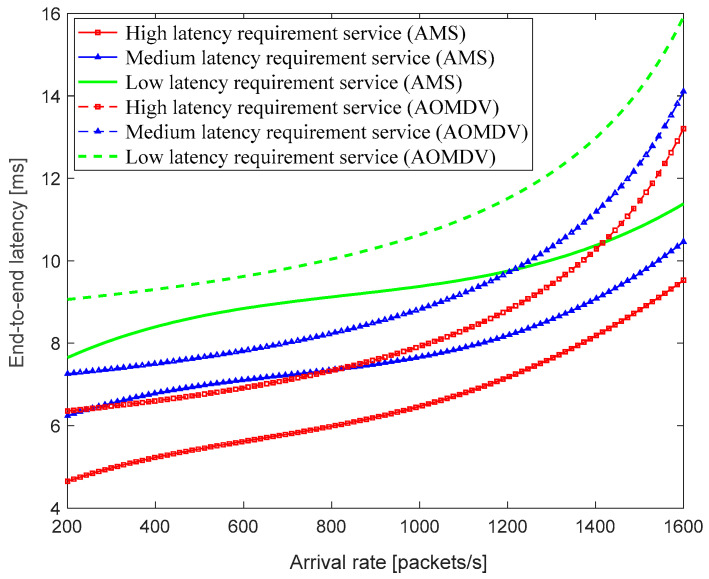
The relationship of end-to-end latency with the arrival rate.

In this simulation, packet No. 1 represents the high-latency requirement service, such as train operation control (OCS), packet No. 2 represents the medium-latency requirement service, such as train operation status monitoring (TOSM), and packet No. 3 represents the low-latency requirement service, such as passenger multimedia service (PMS). The simulations are compared using the traditional AOMDV algorithm and AMS algorithm to transmit these three types of services separately, and the simulation results of the end-to-end latency are shown in Figure 8.

From Figure 8, it can be seen that the multipath selection algorithm with adaptive adjustment of the number of paths based on the service latency requirements can reduce the impact of the arrival rate on the end-to-end latency within a certain extent, and the average end-to-end latency of transmitting the same service data with the AMS algorithm is lower. Based on the data statistics, the average end-to-end latency of the proposed AMS routing algorithm is 28.48% lower than that of the traditional AOMDV routing algorithm. However, when the arrival rate keeps increasing, the average end-to-end latency also increases exponentially.

**Figure 9:**
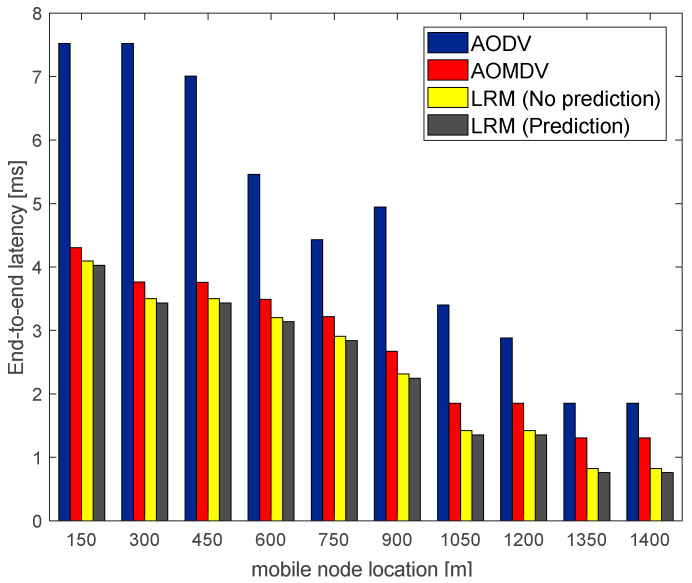
Average end-to-end latency in a good network environment.

**Figure 10:**
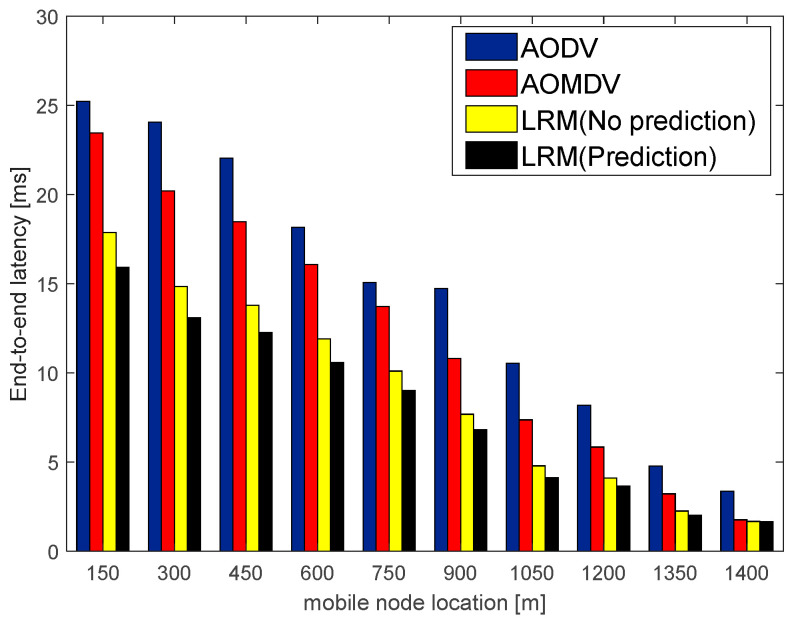
Average end-to-end latency in a bad network environment.

With the AMS algorithm, this paper also employs the local route maintenance scheme and compares the average end-to-end latency using the LRM scheme and the traditional AOMDV, which uses the alternate path repair scheme under different network quality environments. The simulation results are shown in Figure 9 and Figure 10.

Figure 9 and Figure 10, respectively, describe the variation in the average end-to-end latency of the mobile nodes during their movement toward the station sink node in good and bad network environments. From the figures, it can be seen that the average end-to-end latency of the AODV routing algorithm is the highest in the case of the same number of packets transmitted. It is because the AODV routing algorithm does not engage in routine maintenance but rather restarts the route discovery to find a new path for retransmission when a link failure is discovered, which consumes a lot of route discovery time. In the case of a poor network environment, the AOMDV routing algorithm also has a high average end-to-end latency, which is because the link failures happen frequently in bad network environments such that alternate paths need to be enabled frequently for retransmission, which consumes a lot of repair time. On the contrary, in the LRM scheme, it is not necessary to go back to the source node to use alternate paths, but to perform local repair with the help of maintenance nodes, the repair latency of this scheme decreases compared to the AOMDV routing repair latency by about 23.81%. Therefore, the LRM scheme can effectively reduce repair latency and improve transmission efficiency.

#### 4.2.2. Packet Loss Rate Results

**Figure 11:**
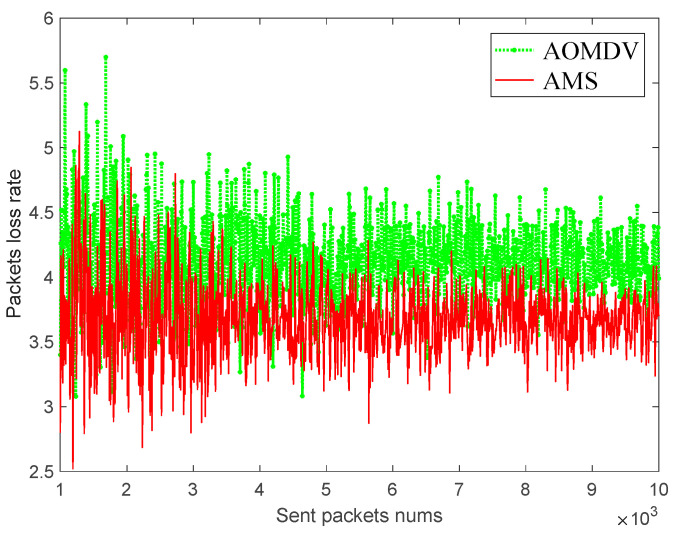
The relationship of packets loss rate with sent packets.

In this section, first, the effect of the traditional AOMDV routing algorithm based on the minimum hop count is simulated and compared with the AMS routing scheme based on the link cost function on the packet loss rate. During the experiments, the path hop count and link quality are considered to have the same degree of contribution to delay and reliability. The simulation results are shown in Figure 11.

As shown in Figure 11, the packet loss rate using the AOMDV routing algorithm is higher than that of the AMS routing algorithm, which is because the path selection based on link cost enables the selection of paths with fewer hop counts and good network quality, reducing the impact of bad path quality on data transmission and improving the success rate of packet delivery.

Second, considering that the route maintenance mechanism can improve the packet loss rate of data transmission, the packet loss rate of traditional AODV routing, traditional AOMDV routing, and AMS routing with the addition of an LRM scheme is simulated and compared, respectively, under different network quality environments. The simulation results are shown in the following figure.

**Figure 12:**
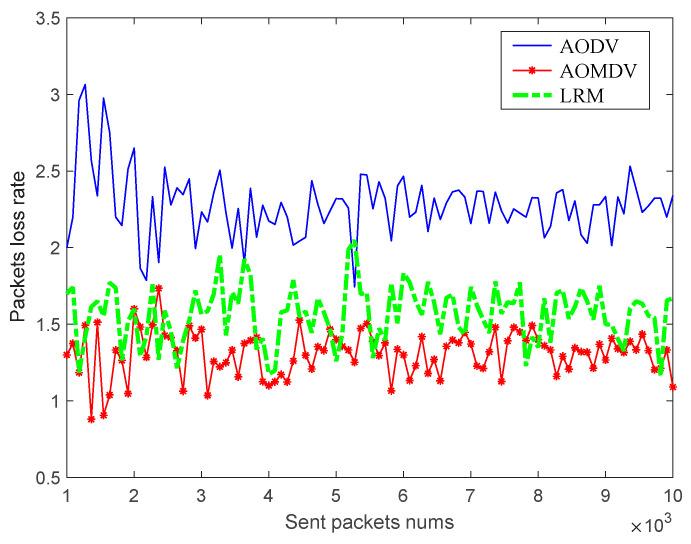
Packets loss rate in a good network environment.

**Figure 13:**
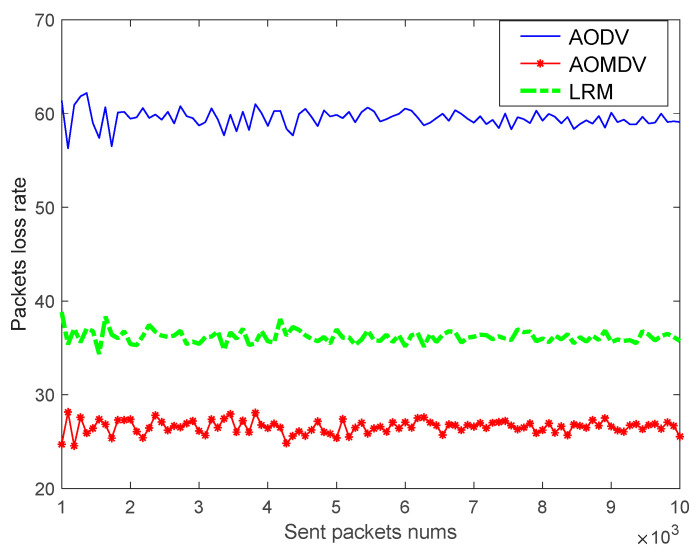
Packets loss rate in a bad network environment.

As shown in Figure 12 and Figure 13, the traditional AODV route is more serious in the packet loss rate due to the lack of a routing maintenance scheme. Using the AOMDV alternate route maintenance mechanism, the lost packets can be retransmitted through multiple alternate paths, which reduces the packet loss rate to a large extent and reduces the effect of the network environment. While the AMS routing based on the LRM scheme has about the same packet loss rate as AOMDV routing when the network quality is good, its packet loss rate will be higher than that of AOMDV routing in a poor network quality environment, which is because the temporary path established by the maintenance node cannot improve the quality of the whole path. However, considering the high maintenance cost and long repair time of the AOMDV routing maintenance scheme, the LRM scheme can reduce the cost of the alternate paths and enable a reduction in the repair time. On the whole consideration, the AMS routing with the LRM scheme has a better performance. In addition, using a small number of alternate paths to ensure high-reliability requirement services and using the LRM scheme for the rest of the services can be considered in future studies.

#### 4.2.3. Throughput Results

**Figure 14:**
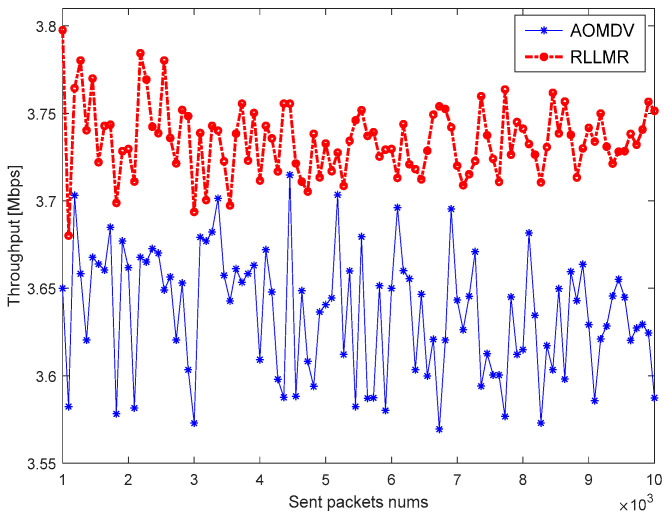
Throughput in a good network environment.

**Figure 15:**
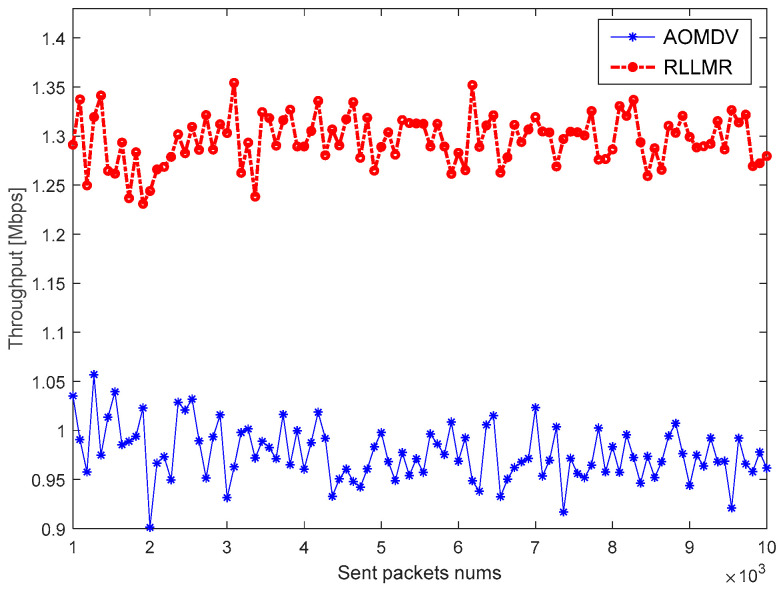
Throughput in a bad network environment.

Throughput means the average rate at which data packets successfully reach the destination node from the source node. In this section, the throughput is simulated under different routing protocols based on the difference in network environment quality, and the simulation results are shown in Figure 14 and Figure 15.

As shown in the figure, the RLLMR algorithm based on AMS and LRM proposed in this paper has a higher throughput compared with traditional AOMDV. The reason is that the packet loss rate of AOMDV based on the alternate path is the lowest, but its repair time is longer, while the LRM-based multipath routing algorithm, although inferior to AOMDV in terms of the packet loss rate, reduces the route maintenance latency significantly. Therefore, the RLLMR algorithm can improve the throughput of the system.

## 5. Discussion

Through the simulation experiments conducted in Section 4, it can be found that the RLLMR algorithm proposed in this paper performs better in terms of latency, reliability, and throughput than AODV and AOMDV.

First, during the route discovery process, AODV and AOMDV find the destination node through the multi hop transmission of RREQ. This process only occurs when needed, saving link overhead. However, due to multi hop transmission, the route discovery time is long. The RLLMR algorithm configures the routing information in the trackside node before the run begins, allowing trains to obtain destination node routing information from wayside nodes, greatly reducing the latency of route discovery. However, this method requires a certain amount of link overhead, so the link overhead will be greater than that of AODV and AOMDV.

Second, in the routing process, both AODV and AOMDV choose paths based on the minimum number of hops, but AODV only selects one path for data transmission, which is easily affected by network congestion, resulting in a high end-to-end time delay and decreased packet delivery rate. AOMDV reduces the link load and reduces the impact of network congestion by using multiple paths for transmission, thereby improving transmission efficiency. In addition, AOMDV also reserves backup paths, which can be used for retransmission when data packets are lost, improving the packet delivery rate. The RLLMR algorithm draws inspiration from AOMDV but differs from AOMDV in that it selects different numbers of paths for transmission based on different latency requirements of services and takes into account two factors: hop count and link quality. By selecting paths with a small hop count and good link quality, the packet delivery rate is improved. In fact, the more transmission paths there are, the greater the transmission efficiency is. However, AOMDV uses the same number of paths for both low-latency and high-latency services, and it may waste path resources or may not meet the latency requirements of the services. Therefore, the RLLMR adaptive path selection method can effectively meet the latency requirements of different services.

Third, AODV does not have a repair plan for packet loss, resulting in a high packet loss rate. As mentioned earlier, AOMDV will retransmit through an alternate path, but enabling an alternate path will result in data needing to be resent from the source node, which takes a long time. In RLLMR, the maintenance node is used to directly retransmit data packets at the current node without returning to the source node. Therefore, this method shortens the routing repair time. However, the repair solution in RLLMR reduces the packet delivery rate in harsh network environments due to the congestion of maintenance nodes due to the need for too many nodes to rely on maintenance nodes. Although RLLMR has a lower packet loss rate than AOMDV in harsh environments, its routing repair time is faster than that of AOMDV. In addition, the throughput is related to the packet loss rate and latency, and the results indicate that the throughput of RLLMR is higher than that of AOMDV. Overall, the performance of RLLMR is superior to that of AOMDV and AODV.

In fact, the RLLMR method proposed in this paper has many limitations. First, this method is only applicable in network scenarios where there are a large number of static nodes and the destination node location is clear. If there are a large number of dynamic nodes, static configuration strategies cannot be implemented, resulting in an increase in routing discovery latency. Second, this method is only applicable when the mobile node prioritizes sending requests to the ground node to establish a wireless communication link and cannot be prioritized by the ground node to send requests to the mobile node because the train is moving and the ground cannot obtain the train’s position information first. Finally, this method currently only considers the situation where a monorail train is heading towards a station, without worrying about energy distribution.

## 6. Conclusions

In this paper, an RLLMR algorithm is proposed to address the problem of traditional on-demand ad hoc routing to meet low-latency and high-reliability vehicle–ground communication requirements. The RLLMR contains static configuration, the AMS algorithm, and the LRM scheme. Static route configuration solves the problem of a long multipath route discovery time, the AMS algorithm adaptively adjusts the number of parallel paths according to the latency requirements of different services to improve the transmission efficiency, and the LRM scheme reduces the maintenance cost by replacing the alternate path with maintenance nodes and shortens the repair latency by establishing temporary paths with maintenance nodes. Finally, the RLLMR algorithm is simulated and compared with the traditional AODV and AOMDV. The results show that the RLLMR algorithm for urban rail transit ad hoc networks can reduce the latency and packet loss rate, and it is suitable for the next-generation urban rail transit vehicle–ground communication system, which carries several services.

## Figures and Tables

**Table 1:** A comparison of routing protocols.

Parameters	AODV	AOMDV	RLLMR
protocol type	on-demand	on-demand	hybrid
discovery latency	long time	long long time	short time
repair latency	NO	long time	short time
reliability	average	excellent	slightly worse than AOMDV
throughput	fair	better than AODV	better than AOMDV
route repair method	NO	alternate path repair	local routing repair
route selection	minimum hop count	minimum hop count	low hop count and good quality

**Table 3:** Simulation parameters.

Parameter	Value
Network size	1500 m × 10 m × 8 m
Communication range	250 m
Carrier frequency	2.4 GHz
Maximum speed	120 km/h
No. 1 and No. 2 Data packet sizes	512 bit
No. 3 Data packet sizes	2048 bit
Transmit Power	30 dBm
Receiver Sensitivity	−70 dBm
(Tx/Rx) Antenna Gain	5 dBi
Mobile node start location	0 m
Station start location	1500 m

## Data Availability

Due to the fact that the data in this paper will continue to be used in the future, the participants of this study did not allow data to be publicly shared.
